# Prospective, randomized, double-blind trial to investigate the efficacy and safety of corneal cross-linking to halt the progression of keratoconus

**DOI:** 10.1186/s12886-015-0070-7

**Published:** 2015-07-21

**Authors:** Stefan J. Lang, Elisabeth M. Messmer, Gerd Geerling, Marc J. Mackert, Tobias Brunner, Sylvia Dollak, Borislav Kutchoukov, Daniel Böhringer, Thomas Reinhard, Philip Maier

**Affiliations:** Eye Center, Albert-Ludwigs-University of Freiburg, Killianstr. 5, 79106 Freiburg, Germany; Department of Ophthalmology, Ludwig-Maximilians-University, Munich, Germany; Department of Ophthalmology, University Hospital Würzburg, Würzburg, Germany; Department of Ophthalmology, Heinrich-Heine-University, Düsseldorf, Germany; Department of Ophthalmology, University Hospital “Alexandrovska”, Sofia, Bulgaria

## Abstract

**Background:**

Corneal cross-linking is widely used to treat keratoconus. However, to date, only limited data from randomized trials support its efficacy.

**Methods:**

The efficacy and safety of corneal cross-linking for halting progression of keratoconus were investigated in a prospective, randomized, blinded, placebo controlled, multicentre trial. Twenty-nine keratoconus patients were randomized in three trial centres. The mean age at inclusion was 28 years. Longitudinal changes in corneal refraction were assessed by linear regression. The best corrected visual acuity, surface defects and corneal inflammation were also assessed. These data were analysed with a multifactorial linear regression model.

**Results:**

A total of 15 eyes were randomized to the treatment and 14 to the control group. Follow-up averaged 1098 days. Corneal refractive power decreased on average (+/−standard deviation) by 0.35 +/− 0.58 dioptres/year in the treatment group. The controls showed an increase of 0.11 +/− 0.61 dioptres/year. This difference was statistically significant (*p* = 0.02).

**Conclusions:**

Our data suggest that corneal cross-linking is an effective treatment for some patients to halt the progression of keratoconus. However, some of the treated patients still progressed, whereas some untreated controls improved. Therefore, further investigations are necessary to decide which patients require treatment and which do not.

**Trial registration:**

NCT00626717, Date of registration: February 20, 2008.

## Background

Keratoconus is a progressive corneal disease that leads to alterations in the overall shape of the cornea and eventual thinning and scarring, with subsequent decreases in vision [1]. The genetic and environmental contributions to the pathogenesis of keratoconus remain controversial [2, 3]. For example, associations with genetic disorders like Down’s syndrome [4] and influences of atopic dermatitis and eye rubbing have been described [5]. Keratoconus usually starts during adolescence and progresses until the third or fourth decade of life [6, 7]. The CLEK (Collaborative Longitudinal Evaluation of Keratoconus) Study [8] showed a mean change in flatter keratometry readings of 1.6 dioptres in the natural course of keratoconus progression over an eight year period, where higher rates of progression occurred in younger than in older patients. Furthermore, an increase of more than 3 dioptres in spherical equivalent was observed in 24.1 % of patients with risk factors for high progression, such as young age and poor high-contrast visual acuity [8].

The intention of the corneal cross-linking (CXL) procedure using riboflavin is to halt the progression of keratoconus. The induction of covalent molecular cross-links in corneal tissue using riboflavin and UVA-Radiation was first described by Spoerl et al. in porcine corneas in 1998 [9]. In vitro experiments have since shown that CXL leads to changes in the thermo-mechanical behaviour of the cornea [10], the collagen fibre diameter [11, 12], the resistance to enzymatic digestion [13] and the corneal thickness [14]. In addition, apoptosis and loss of keratocytes have been observed [15].

The method was clinically introduced in 2003 with a non-randomized pilot study in 22 patients [16]. In this prospective pilot study, Wollensak et al. reported a halt in the progression in all treated eyes [16]. Since then, many more non-randomized studies, case series or cohort studies [17–23] have demonstrated similar results, with the largest trial being that of Raiskup-Wolf et al., which included 241 eyes [18].

Four promising randomized controlled trials of corneal cross-linking were performed in the past. Wittig-Silva et al. first published interim results of an Australian trial in 2008 which showed a stabilization of all treated eyes [24]. The final results with 46 patients in the treatment group and 48 patients in the control group demonstrated an improvement in maximal keratometric power (Kmax) and visual acuity in the treated patients, while the untreated patients showed further keratoconus progression [25]. A second randomized controlled trial performed by Hersh et al. included eyes with keratoconus and post-lasik ectasia [26], as well as a sham treatment group that received corneal cross-linking after three months. All patients were aware of their randomly assigned groups. An improvement in uncorrected and corrected visual acuity, as well as the topographic measurements was reported in the treatment group. After one-year follow-up, an overall improvement in corneal shape was observed [27]. A third randomized controlled study, conducted by O’Brart in 24 patients, demonstrated an improvement in in corrected visual acuity, Orbscan simulated and keratometry simulated astigmatism [28]. The control group consisted of the fellow eyes. Sharma et al. performed the fourth study—a prospective randomized controlled trial in an Asian population with a total of 43 patients. A decrease was observed in the maximum and minimum keratometry in the cross-linking group in this study [29].

The safety of cross-linking has also been assessed in various trials. The removal of the epithelium can lead to the occurrence of bacterial keratitis [30], corneal melting [31], haze [32], corneal endothelial loss [33] and even calcific band keratopathy [34]. A study by Greenstein et al. described an increase in haze up to three months after treatment, followed by a decrease up to month 12 [32]. Koller et al. described an overall complication rate of 2.9 % in a prospective trial and identified risk factors such as patient age of more than 35 years and a visual acuity better than 20/25 [35].

The efficacy and safety of corneal cross-linking has been suggested by different authors, but clear proof of a therapeutic effect is not available through a placebo-controlled study with an independent control group [36]. Therefore, we investigated the efficacy of corneal cross-linking with riboflavin in halting the progression of keratoconus by conducting a placebo-controlled, randomized, blinded, multicentric clinical trial that included an independent control group.

## Methods

This study was performed at three university eye hospitals—in Freiburg, Munich and Würzburg—and was registered at cliniclatrials.gov (NCT00626717). Ethics Committee approval was obtained at Albert-Ludwigs-University of Freiburg, Ludwig-Maximilians-University, Munich and University Hospital Würzburg. Written informed consent was obtained from the patient, or in case of minors, from the parent or legal guardian. Research adhered to the tenets of the Declaration of Helsinki.

### Inclusion and exclusion criteria

The inclusion criteria for this study were keratoconus at an early stage, defined as correction of refractive error possible with spectacles or contact lenses. The progression had to be either proven by measurement of the corneal topography (an increase of more than 1 dioptre in Kmax within one year) or by a clinically significant change in refraction. The change in refraction was defined as a change in spectacle correction or change in contact lens parameters. Exclusion criteria were patient age under 12 years, corneal thickness below 450 μm, further pre-existing ocular diseases, prior ocular surgery, pregnancy and allergy to riboflavin. Written informed consent was obtained prior to participation.

The initial examination included best corrected visual acuity, slit lamp examination and ophthalmoscopy and assessment of corneal topography with the Orbscan II system (Bausch & Lomb) at the Freiburg centre or with the Pentacam (OCULUS Optikgeräte GmbH) at the Munich and Würzburg centres. The change in keratometric corneal refraction was the primary evaluation and every measurement on each patient was done using the same topography system; consequently, we only assessed the longitudinal changes. Systematic errors due to the keratometry measurements by different systems are therefore unlikely. Computerized randomization was performed at the coordinating centre in Freiburg. Randomization was stratified by centre and the randomizations were submitted via fax. The patients were either randomized to the treatment or the placebo group. On the same day, the worse eye was treated either with a standard CXL protocol [16] or with a sham procedure. The worse eye was defined as the eye with a greater progression, or steeper K-values in case of equal progression in both eyes. The CXL was performed according to an established protocol, as follows: corneal epithelial removal, 0.1 % riboflavin eye drops (MedioCROSS H Riboflavin > 0,1 %, Medio-Haus Medizinprodukte GmbH, Kiel) every two minutes for 30 min, and UVA 370 nm at 3 mW/cm^2^ for 30 min with continued application of 0.1 % riboflavin eye drops every two minutes. The sham procedure consisted of application of fluorescein eye drops every two minutes for 30 min, radiation with visible blue light for 30 min and no epithelial removal.

### Follow-up

Postoperative slit-lamp examinations were scheduled at days 1, 3, 5 and 7 after the intervention. Examinations included best corrected visual acuity and slit lamp examination, as well as ophthalmoscopy and corneal topography. Topography assessment was repeated three times each visit and the averaged K-values were entered into the case report forms. Examination was performed on days 14, 30 and 90 as well as months 6, 9, 12, 18, 24 and 36 post intervention.

The controls were easily identifiable during the first 4 visits since the epithelium was not removed. Therefore, a second examiner, who had not participated in the treatment or the first examinations, took over follow-up examinations from the 5^th^ visit on to achieve a blinding of the examiner. The patients were informed about possible symptoms of dry eye and pain due to epithelial removal. However, patients were not informed about the connection of these symptoms with the placebo or CXL treatment. The patients therefore were not fully aware of their assignment to the placebo or CXL group. The use of contact lenses was not restricted.

### Clinical endpoints and statistics

The primary end-point was progression of keratoconus. An increase of 1 dioptre per year in patients younger than 20 years and an increase of 0.2 dioptres per year in the complete cohort was considered as progression according to the data of the natural course of the disease from the CLEK Study. This was measured by the longitudinal change in keratometric corneal refraction (maximum simulated K-readings) and calculated by linear regression over the steeper K-readings plotted against follow-up time for each patient. Sample size was calculated to ensure detection of a halting of the spontaneous progression rate from the CLEK study [37]. According to the CLEK study, we assumed a progression risk of 30 % within two years. A one-year recruiting time and minimum follow up of two years were expected. A two-sample *t*-test power calculation estimated a statistical power of 80 % with a total sample size of 65 patients per group. Unfortunately, this was not achieved, since most candidates were either non-progressive or reluctant to undergo randomization. Therefore, we closed the trial after recruitment of 30 patients. As secondary endpoints, we also assessed the minimal simulated K-readings, the central corneal thickness, worsening of best corrected visual acuity and the occurrence of further adverse events. The trial was originally analysed according to the intention-to-treat principle. Because we did not stratify randomization for age in the protocol, we opted for a multiple linear regression model with group assignment and age at inclusion to control for these potential confounders.

## Results

**Fig. 1:**
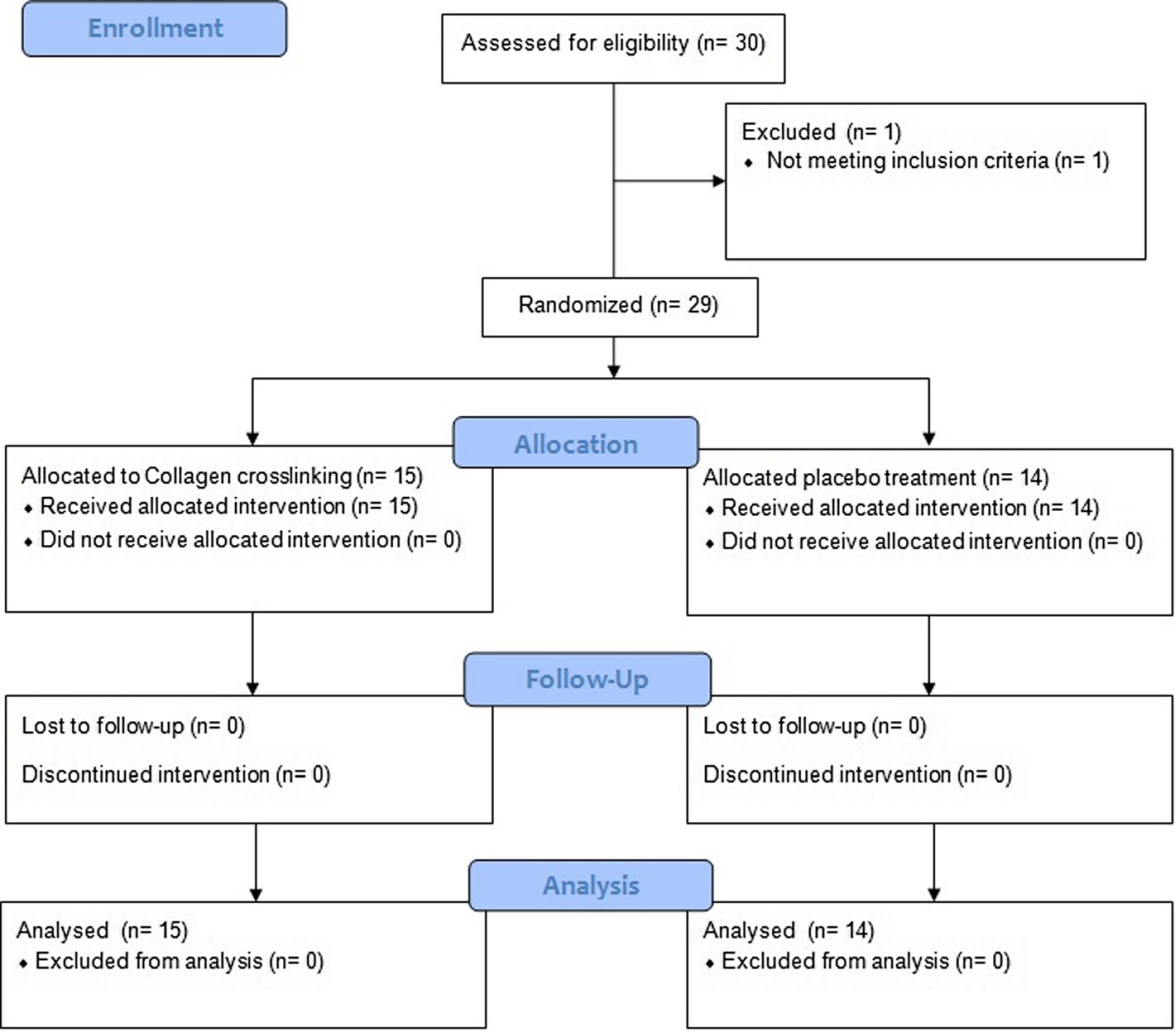
Consort flow-chart. Three patients had an incomplete follow-up of one year. The data were included in the analysis. One patient belonged to the treatment arm, the other two to the placebo group

The consort chart is depicted in Fig. 1. One patient had to be excluded prior to randomization since the inclusion criteria were not fulfilled, leaving 29 patients available for analysis. Of these, 15 patients had documented progression of keratoconus; the remaining 14 patients had reported visual deterioration or worsening of spectacle refraction. The mean age at inclusion was 28 years (median range: 17 to 53). In total, 15 patients were randomized to the treatment and 14 to the control group. Follow-up averaged 1098 (quartiles 802 to 1131) days. Baseline and follow-up characteristics of the treatment and control group are summarized in Table 1. Three patients had an incomplete follow-up and did not participate in further examinations after one year; one of these three belonged to the treatment arm, the other two to the placebo group.

**Table 1:** Baseline characteristics of the placebo and treatment (CXL) groups

		Placebo group (*N* = 14)	CXL group (*N* = 15)	
	Female Patients	14 % (2)	27 % (4)	*p* = 0.41
	Age at inclusion	25.8 +/−7.4	29.5 +/−11.1	*p* = 0.55
Centre:	Freiburg	64 % (9)	40 % (6)	*p* = 0.10
	München	36 % (5)	33 % (5)	
	Würzburg	0 % (0)	27 % (4)	
	Corneal thickness at inclusion	468.8 +/−25.4	466.8 +/−27.8	*p* = 0.91
	Kmin at inclusion	46.5 +/−4.3	44.0 +/−1.7	*p* = 0.07
	Kmax at inclusion	50.9 +/−5.7	47.3 +/−2.2	*p* = 0.05
	Visual acuity at inclusion (logMAR)	0.39 +/−0.37	0.25 +/−0.15	*p* = 0.38

**Fig. 2:**
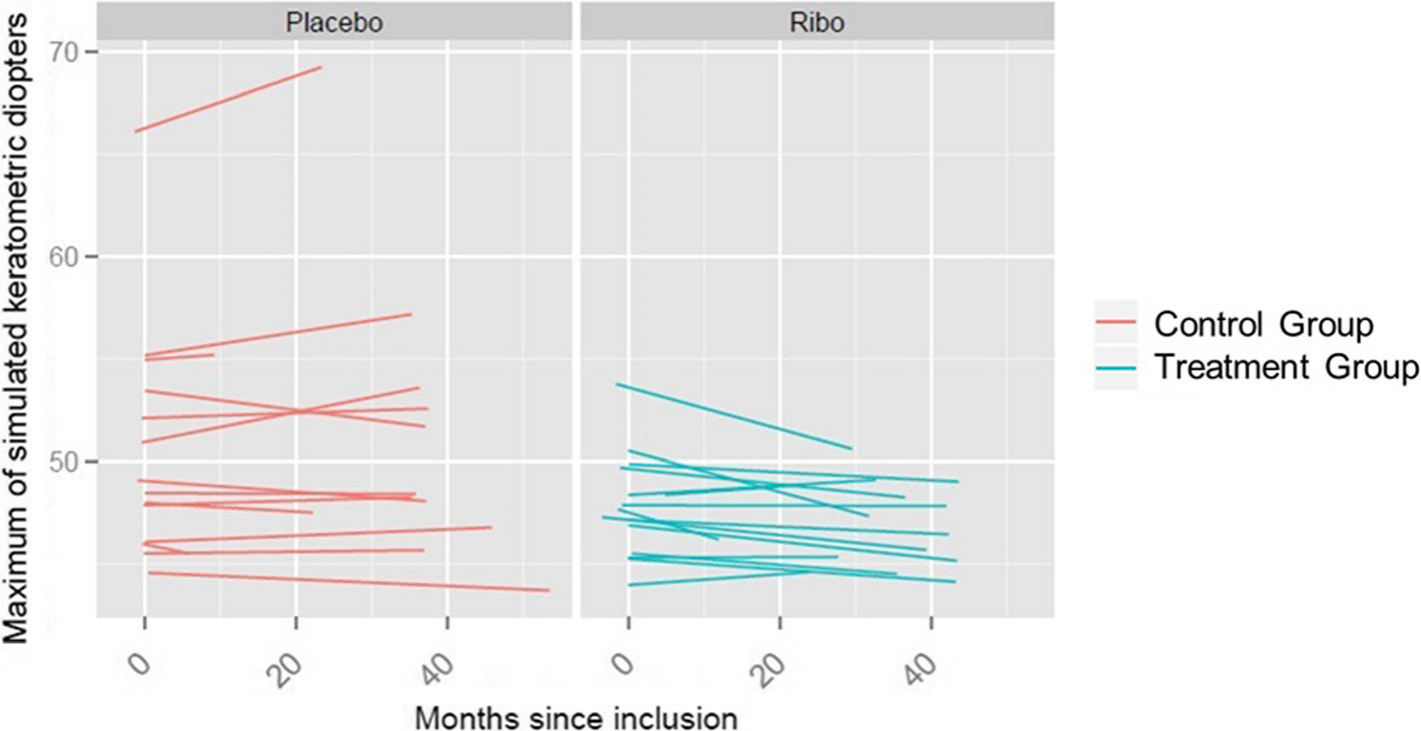
Corneal refractive power for each patient over the time of the follow-up

Four patients in the treatment group showed a slight steepening of their corneal topography. Eleven patients showed a slight flattening or remained stable. In the control group, eight patients showed steepening and six patients showed flattening of the simulated maximum K-reading (Table 1, Fig. 2). In the treatment group the corneal refractive power (Kmax) decreased in mean (+/−standard deviation) by 0.35 +/− 0.58 dioptres per year. The control-group showed an increase of 0.11 +/− 0.61 dioptres per year (Fig. 2). This difference was statistically significant (*p* = 0.02) in the multiple linear regression model. Considering our definition of progression, two patients in the placebo group and one patient in the treatment group showed a clinically significant progression. The corneal refractive power showed an increase of 1.5 dioptres per year in a 38-year-old patient.

The preoperative central corneal thickness was 468 +/− 25.36 μm in the control group and 466 +/− 27.76 μm in the treatment group. Three years after the intervention, the control group corneal thickness was 467 +/− 23.95 μm and the treatment group thickness was 449 +/−71.96 μm; this difference was not statistically significant. All secondary endpoints are summarized in Table 2.

**Table 2:** Secondary endpoints: corneal thickness, maximal and minimal simulated K-readings and visual acuity in the placebo and treatment (CXL) group at the end of the trial

	Placebo group	CXL group	
Corneal thickness (μm) at the end of follow-up	467.3 +/−24	449.2 +/−72	*p* = 0.96
Kmin (dpt) at the end of follow-up	46.1 +/−4.7	43.5 +/−1.7	*p* = 0.59
Kmax (dpt) at the end of follow-up	51.2 +/−6.9	46.9 +/−2.1	*p* = 0.59
Visual acuity (logMAR) at the end of follow-up	0.23 +/−0.27	0.22 +/−0.14	*p* = 0.61

None of the patients sustained bacterial keratitis or subepithelial infiltrates or had to undergo any kind of surgery after the intervention.

During the complete follow-up period, eight patients experienced a significant worsening of the best corrected visual acuity: four in the control group and four in the treatment group (Table 3). Chi-square test showed no statistical difference.

**Table 3:** Adverse events in both groups. The treatment (CXL) group showed significantly more haze and corneal erosion during the follow-up period. After 3 years, all but 3 eyes showed a complete resolution of the haze

		Placebo group	CXL group	
Maximal haze	0	71 % (10)	0 % (0)	*p* < 0.01
	1	7 % (1)	20 % (3)	
	2	21 % (3)	73 % (11)	
	3	0 % (0)	7 % (1)	
Corneal erosions	0	79 % (11)	7 % (1)	*p* < 0.01
	1	0 % (0)	7 % (1)	
	2	7 % (1)	13 % (2)	
	3	7 % (1)	60 % (9)	
	4	7 % (1)	13 % (2)	

All eyes in the treatment group had postoperative epithelial defects due to the epithelial removal during surgery. In the placebo group, three eyes developed epithelial defects after the intervention probably due to drying of the ocular surface despite intensive eye drop application during the process. The treatment group showed significantly more haze (15 of 15 patients) than was observed in the control group (four of 15 patients, p < 0.001, Table 1). After three years, all but three eyes showed a complete resolution of the haze (Table 3).

## Discussion

A limitation of our study is its sample size, which was considerably smaller than planned. The patients were increasingly unwilling to be randomized against sham procedures. We eventually had to terminate the recruitment period after recruitment stalled. For this reason, our study includes only 30 patients and not the anticipated number of 120. However, we strongly believe that our results are of interest because equally-sized unbiased data can no longer be collected these days.

This is one of the first randomized, double-blind, placebo-controlled multicentre clinical trials to investigate the efficacy of riboflavin CXL to halt keratoconus progression. The control group underwent a sham procedure and did not comprise the fellow eyes of the treated patients. This is especially important since keratoconus is an asymmetric disease [38]. The study reported by O’Brart et al. used a control group without sham treatment, consisting of the fellow eye of the 24 study patients. Randomization was performed to account for asymmetric progression. Only three eyes of 24 in the control group showed a slight deterioration in the investigated parameters and the majority of the untreated eyes remained stable [28]. Intra-individual controls, without sham treatment or blinding, were also used by Wittig-Silva et al. [24, 25], whose data showed stabilization of all treated eyes compared to progression in the fellow eyes. The trial by Hersh et al. included a control group that underwent a sham procedure. The patients were aware of their assigned group. Keratoconus in this group remained stable and no significant changes were noted in any of the investigated corneal indices or in best corrected visual acuity. However, a crossover to the treatment group was performed three months after placebo treatment [26]. This group also showed no significant changes in corneal indices or best corrected visual acuity at the time of conversion. A trial by Sharma et al. included a control group with a sham procedure. Epithelial debridement was also performed in the sham group. Similar to our study, this trial showed a significant decrease in Kmax in the cross-linking group. Additional confocal analysis of the epithelial healing and the regeneration of the sub epithelial plexus was performed in this study [29].

Blinding the patients in the present study was challenging due to its design. Patients of the CXL arm were more likely to suffer from postoperative pain in comparison to the controls. To address this, we educated all patients that postoperative pain can occur but not at a 100 % rate. Nevertheless, we cannot fully rule out that some patients were possibly aware of their treatment arm. Our opinion, however, is that this did not introduce any systematic bias.

Despite its low power, the study also confirms that CXL can significantly alter the steepening of the topographic K-readings in keratoconus patients, since the overall maximum K-reading decreased by 0.35 +/− 0.58 dioptres per year. This finding was adjusted for age, a well-known risk factor for progression of keratoconus. However, four of the 15 patients in the treatment group showed an increase in Kmax from 0.02 to 0.32 dioptres per year. The age of these patients ranged from 19 to 38 years. Six of 14 patients in the control group showed no steepening of any kind in their corneal topographies. Moreover, the remaining eight patients in this group all suffered only from a mild to moderate steepening of the topographic K-readings, so that only two of the controls finally fulfilled our criteria for the progression of keratoconus. Incidentally, one of these two patients had the highest slope and by far the highest simulated maximum K-reading of more than 65 dioptres. Since this patient was assigned to the control group, this might represent a bias in our study. However, omission of this patient from the statistical analysis still reveals a significant difference between both groups (data not shown).

In the treatment group, four patients showed a slight steepening of their corneal topographies. One of these patients fulfilled our criteria for the progression of keratoconus. The reason why only a few of the control patients maintained progression during the course of the trial might be that, according to the protocol, the progression required for inclusion did not have to be proven by keratometry. Another possible explanation is that the noise of the tomography system that may render the detection signs of progression impossible, especially in patients with higher K-values or irregularities of the surface [25, 39–41] or after use of contact lenses. We sought to counteract this potential error by repeating the measurements on each visit. Nevertheless, a small inaccuracy of the keratometric K-readings cannot be fully ruled out. The recent consensus states that the progression of keratoconus is best evaluated with additional thickness and posterior keratometric data [42]; however, this had not been established at the time of preparation of the study protocol.

Some of the patients may have had a progression rate that was lower than estimated. This limitation of our study may be due to the fact that the patients did not require a proven keratometric progression for enrolment in the trial.

Not all enrolled patients had documented objective progression. We therefore characterized this subgroup to rule out confounding from this factor. Fifteen patients were enrolled with objective documented progression, whereas 14 patients only had subjective worsening. Of those 14 patients, eight were in the treatment and six were in the placebo group. The difference in slope of Kmax was not statistically significant (*p* = 0.47) between the patients with objective progression before recruitment (−0.22 +/− 0.57 dioptres per year) and the patients with subjective worsening (−0.03 +/−0.67 dioptres per year). We added this possible confounder to the multiple linear regression model. This factor missed statistical significance (*p* = 0.39) and the treatment effect remained statistically significant (*p* = 0.02).

The patient age may also account for the low progression rate observed in the study, especially since nine patients were above 30 years of age. This age group has been shown to have only subtle changes in keratometry [37]. However, this was also true for the treatment group as a principle of randomization. The low rate of progression is incidentally concordant with the CLEK Study and other CXL trials [24, 26, 28, 32]. Nevertheless, our study found a slight, but statistically significant, superiority of CXL over placebo treatment regarding the change in corneal topography. This agrees with the findings of the other prospective trials [24, 26, 27]. The absence of progression in many untreated patients emphasises the need for careful selection of suitable patients in order to prevent overtreatment.

Since three patients were lost to follow-up, a higher incidence of progression cannot be fully ruled out.

The change in visual acuity did not show a statistically significant difference between the control and treatment group. Even though the cross-linking group experienced more haze, this obviously did not impair the visual acuity of the patients, as also found by Greenstein et al. [32]. The occurrence of haze after corneal cross-linking has been described by several authors [32, 34]. The decrease in haze over a period of several months to years is also confirmed. Three patients had remaining haze three years after corneal cross-linking, but none of these patients suffered any visual loss in comparison to their vision before treatment. The absence of other more severe complications shows that corneal cross-linking is a safe therapeutic option for keratoconus.

## Conclusions

Our randomized trial confirms a beneficial effect of CXL with riboflavin and UVA radiation as a treatment for progressive keratoconus that halts the changes in corneal topography. We also found that some patients show no worsening or progression of any kind, even without treatment, and we observed a slight steepening in maximum K-readings despite CXL. The number of patients needing treatment may therefore be higher than expected. Some patients might receive an overtreatment if corneal cross-linking was performed and CXL seems to be less effective in some patients than in others. Therefore, in the future, we need to determine the clinical parameters that will allow for a distinction of keratoconus patients who will benefit from the treatment and the ones who will not.

## Abbreviations

CLEK: Collaborative Longitudinal Evaluation of Keratoconus
CXL: Riboflavin corneal cross-linking
Kmax: Maximal keratometric power
